# Navigating the Unexpected: Iatrogenic Aortic Injuries during Transcatheter Aortic Valve Replacement (TAVR)

**DOI:** 10.3390/jcm12247630

**Published:** 2023-12-12

**Authors:** Shaelyn Cavanaugh, Hossein Amirjamshidi, Kazuhiro Hisamoto

**Affiliations:** Division of Cardiac Surgery, Department of Surgery, University of Rochester Medical Center, Rochester, NY 14642, USA; shaelyn_cavanaugh@urmc.rochester.edu (S.C.);

**Keywords:** iatrogenic aortic dissection, TAVR, type A dissection

## Abstract

The introduction of transcatheter aortic valve replacement (TAVR) has undeniably changed the landscape of valvular heart disease management over the last two decades. A reduction in complications through improvements in techniques, experience, and technology has established TAVR as a safe and effective alternative to surgical aortic valve replacement. However, it is important to consider the potential risks associated with TAVR and ways in which life-threatening complications can be identified and managed in a timely fashion. In this article, we review some catastrophic iatrogenic aortic injuries that are described in the literature and present a case of an acute iatrogenic type A aortic dissection that occurred during a transcatheter aortic valve replacement (TAVR). After valve deployment, a routine neurologic examination noted the new onset of a left-sided facial droop and upper extremity weakness. Urgent imaging revealed an extensive type A aortic dissection, and the patient was taken to the operating room for surgical repair. The coordination of our multidisciplinary team allowed for prompt recognition of her neurologic symptoms, urgent imaging, and timely transport to the operating room, all of which contributed to the successful management of this life-threatening procedural complication.

## 1. Introduction

Transcatheter aortic valve replacement (TAVR) has been increasingly utilized over the last decade for patients with high and intermediate risk of perioperative morbidity and mortality and is now an option for patients in all risk categories. Despite the benefits of transcatheter interventions, there are some rare complications that can be life-threatening. The management of complications in these patient populations is challenging due to both the acuity of the complication and the patients’ underlying comorbidities, which often precluded them from open surgery in the first place. This is especially relevant to cases of aortic injury during TAVR, which include annular rupture, hematoma, aortic-ventricular and aortic-atrial fistulas, and aortic dissections. Here, we will review some of the potential risk factors, clinical characteristics, and outcomes of TAVR-associated aortic injuries, with a specific focus on type A aortic dissections. To highlight the role of a successful team-based approach, we will present a representative case of a catastrophic aortic injury and how this injury was successfully managed with conversion to open surgical repair.

## 2. Detailed Case Description

A 75-year-old female patient developed progressive exertional dyspnea. She was a former smoker with a history of: asthma, for which she was a long-term user of prednisone; obstructive sleep apnea, for which she used CPAP; type 2 diabetes mellitus; chronic kidney disease; hypertension; and hyperlipidemia. A transthoracic echocardiogram revealed severe aortic stenosis with a peak velocity of 4.7 m/s, a mean gradient of 57 mmHg, a peak gradient of 83 mmHg, and an aortic valve area of 0.59 cm^2^ with a severe reduction in leaflet mobility and no significant aortic regurgitation. The patient had an intermediate Society of Thoracic Surgeons risk of mortality for isolated surgical aortic valve replacement (SAVR) [1]. Based on patient preference and heart-team discussion, the decision was made to proceed with TAVR. During the workup for TAVR, the patient unfortunately developed the COVID-19 infection and was subsequently hospitalized with pneumonia and bilateral segmental pulmonary emboli. The patient was placed on warfarin anticoagulation for treatment of her pulmonary emboli. After successful treatment and recovery from these acute pulmonary illnesses, the patient was re-evaluated by our multidisciplinary team, and the decision was made to move forward with TAVR, given her ongoing symptoms of severe aortic stenosis. Her anticoagulation regimen was transitioned to low-molecular-weight heparin, which was held for two days prior to the procedure. Preoperative chest CT did not reveal any abnormalities of the aorta, including entry tears, aneurysmal dilation, or significant calcifications. The imaging revealed a calcified trileaflet valve with an aortic annulus measuring 23.6 and 26.8 mm in short and long diameter, respectively, with an area of 472.7 mm^2^. The sinotubular junction diameter measured 32.3 mm × 28.9 mm, and the maximum ascending aorta diameter measured 34.6 mm × 34.9 mm. The left and right coronary heights measured 22.0 mm and 20.9 mm, respectively. The right and left femoral artery diameters were 6.1 mm × 7.3 mm and 7.6 mm × 8.9 mm, respectively.

**Figure 1:**
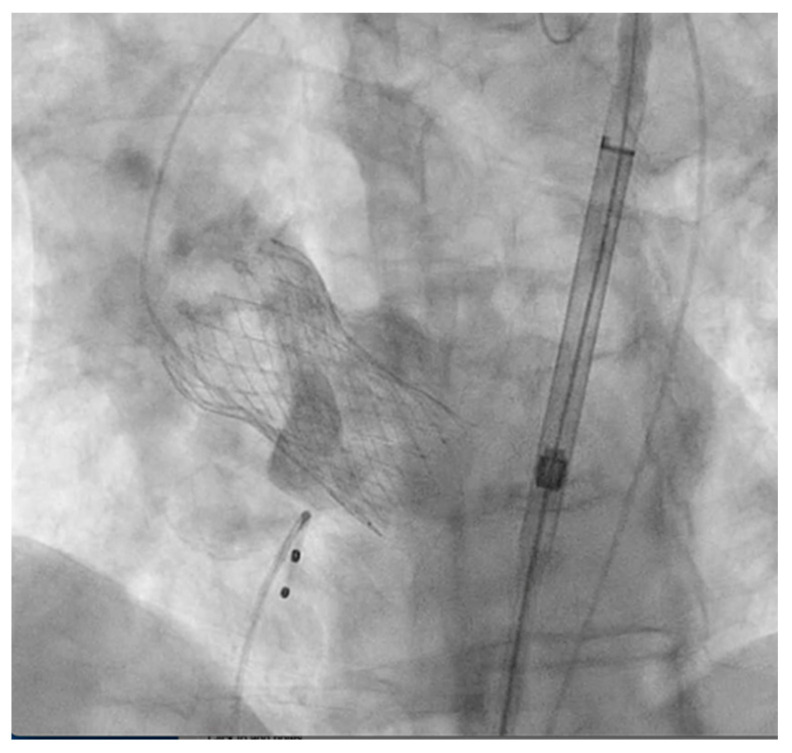
Completion aortography showing no paravalvular leak.

**Figure 2:**
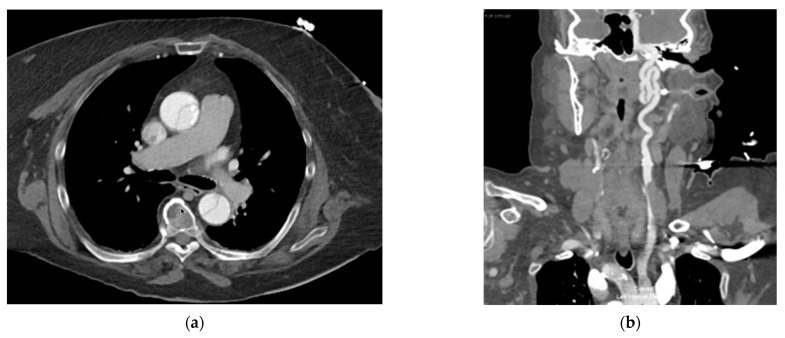
(**a**) Chest CT, demonstrating dissection near the transcatheter heart valve. (**b**) CTA head and neck, demonstrating near-complete occlusion of the right common carotid artery and complete occlusion of the right internal carotid artery.

The patient was brought to the cardiac catheterization lab and placed in the supine position for the TAVR procedure, which was performed under conscious sedation. Pre-dilatation balloon aortic valvuloplasty (BAV) was performed using a 20 mm balloon without any changes in hemodynamic parameters. The patient then underwent a successful placement of a 29 mm Medtronic Evolut FX system via the left trans-femoral approach, which corresponds to 19% oversizing and was chosen based on the preoperative CT measurements described above. There were no arrythmias noted on the EKG, and vital signs remained within normal limits during the procedure. The transthoracic echocardiogram and completion aortography demonstrated no significant paravalvular leak (PVL) and no central aortic regurgitation (Figure 1). However, at the conclusion of the procedure, the patient complained of new-onset chest pain, and a routine neurologic exam revealed a left-sided facial droop and left upper extremity weakness. Given these symptoms, she was taken from the cardiac catheterization lab for an urgent CT angiogram of the head, neck, chest, and abdomen. Imaging unfortunately revealed an extensive aortic dissection originating at the superior margin of the prosthetic valve, extending into the great vessels and upper abdominal aorta to the level of the left common iliac artery (Figure 2). On closer evaluation, there was near-complete occlusion of the right common carotid artery and complete occlusion of the entire cervical and intracranial segments of the right internal carotid artery. The dissection involved the origin of the left common carotid artery, with no dissection identified in the left internal carotid.

Fortunately, the visceral vessels all appeared to originate from the true lumen. Given the extent of the dissection and severity of neurologic symptoms, the patient was urgently taken to the operating room from the CT scan suite after discussion with her family.

The patient was placed on the operating room table in the supine position, and his chest, abdomen, and bilateral legs were prepped and draped in the standard fashion. Following intubation, an intraoperative transesophageal echocardiogram (TEE) was performed, demonstrating normal bioprosthetic valve function and a dissection flap at the level of the sinotubular junction. Bilateral radial arterial lines were placed, revealing equal blood pressure on the right and left. Cerebral oxygen saturation monitoring was utilized. The right axillary artery was exposed, was not affected by the dissection, and was thus used for arterial cannulation using an 8 mm chimney graft. Following peripheral venous cannulation via the right femoral vein, a cardiopulmonary bypass was initiated with immediate elevation of right cerebral oxygen saturation. There was no hemopericardium upon opening the pericardium. A large entry tear was noted in the proximal ascending aorta, with no entry tear immediately visible in the root or the aortic arch. Antegrade and retrograde cerebral perfusion were utilized with 24 °C circulatory arrest. The hemiarch replacement was performed with a 26 mm straight graft. The transcatheter heart valve (THV) was then explanted to allow close inspection of the aortic root for an entry tear. The aortic valve was then replaced with a 23 mm Edwards Inspiris valve (Edwards Lifesciences LLC, Irvine, CA, USA). The patient was weaned from the cardiopulmonary bypass without complications. Postoperative TEE revealed a normal left ventricular ejection fraction and a normal prosthetic aortic valve function with no paravalvular leak. Total cardiopulmonary bypass time was 172 min, total cross clamp time was 135 min, and selective cerebral perfusion time was 28 min.

The patient recovered remarkably well following surgery. The patient was able to follow verbal commands shortly after surgery; however, she remained intubated due to episodic hypoxia secondary to mucous plugging and fluctuating mental status. A postoperative head CT demonstrated multiple subacute infarcts in the right frontal and temporal lobes. Repeat CTA demonstrated improved caliber of the right common and internal carotid arteries, with an area of stenosis at the right internal carotid artery. Given this finding and persistent right-sided weakness, the patient underwent a diagnostic cerebral angiogram. This demonstrated a subtle dissection flap within the right common carotid and internal carotid arteries; however, there was no evidence of flow-limiting stenosis or large vessel occlusion that would be amenable to thrombectomy or stenting. The patient was extubated on postoperative day 5 and made significant functional improvements following her intensive care unit stay. While the patient did have residual left upper extremity weakness and moderate dysphagia, she was able to eventually ambulate with assistance and had minimal cognitive deficits. She was discharged on postoperative day 14 to acute inpatient rehabilitation, where her functional status continued to improve. After further rehabilitation at a skilled nursing outpatient facility, the patient was eventually able to be discharged home. A head CT performed for surveillance one month postoperatively showed regression of the subacute right frontal and temporal lobe infarcts. Recent abdomen and pelvis CT scans revealed a stable appearance of the aortic dissection, with patent visceral branch vessels. The most recent transthoracic echocardiogram demonstrated a normally functioning prosthetic aortic valve without significant stenosis or regurgitation.

## 3. Discussion

The introduction of transcatheter aortic valve replacement (TAVR) has undeniably changed the landscape of valvular heart disease management over the last two decades. Reduction in complications through improvements in techniques, experience, and technology has established TAVR as a safe and effective alternative to surgical aortic valve replacement in specific populations. However, it is important to consider the potential risks associated with TAVR and the ways in which life-threatening complications can be diagnosed and managed in a timely fashion. Although rare, injuries to the aorta during TAVR can be catastrophic.

### 3.1. Aortic Annular Rupture

One of the most frequently observed major aortic complications of TAVR is annular rupture, which can be characterized based on the location of the rupture (intra-annular, sub-annular, supra-annular, and combined). An annular rupture or injury to the aortic root or left ventricular outflow tract can occur during many steps of a TAVR procedure. These include balloon dilation of the native aortic valve, deployment of the prosthetic valve, or post-deployment dilation. Balloon-expandable valves seem to be more commonly associated with annular ruptures during TAVR. However, during the placement of self-expandable valves, annular rupture is typically associated with re-ballooning following implantation when a paravalvular leak is observed [2]. A recent meta-analysis demonstrated a pooled estimated rate of annulus rupture during TAVR to be approximately 0.5% [3]. Several studies have aimed to identify the anatomic, procedural, and patient-related risk factors for annular rupture during TAVR. One study identified moderate and severe LVOT/subannular calcification and prosthesis oversizing of ≥20% as two factors that were significantly associated with aortic root rupture [4]. Some additional presumed risk factors include a small aortic annulus, a narrow aortic root, leaflet calcification, and excessive oversizing [2]. Similar to other iatrogenic aortic injuries during TAVR, annular rupture treatment depends on the extent of the rupture and the severity of clinical manifestations. Some cases have demonstrated successful management using various percutaneous management techniques, including coil embolization [5]. One case report describes a pre-dilation-induced annular rupture that was successfully sealed by proceeding with the planned TAVR valve deployment [6]. Another case report described an aortic annular rupture after valve placement; however, a subsequent valve-in-valve technique with the deployment of a second TAVR valve was successful [7]. Conversely, cases with significant hemodynamic compromise often require surgical bailout, which has been successfully demonstrated in several case reports [8,9]. In a study that examined cases of TAVR that required surgical bailout, the reason for surgical bailout was annular rupture in 14.16% of cases [10]. Based on a study using data from a large TAVR registry, annular rupture was the second most common reason for emergent cardiac surgery during transfemoral TAVR, and annular rupture was observed in 21.2% of patients requiring emergent conversion to surgery. Interestingly, this study demonstrated a decrease in the incidence of this complication over time and postulated that this may reflect the more routine use of CT scan assessment of the aortic root prior to TAVR, and thus more accurate valve sizing and identification of potential anatomic risk factors [11].

### 3.2. Aortic Hematoma

While frequently occurring spontaneously, intramural hematomas are another possible iatrogenic aortic complication of TAVR. One case report described a case of an intramural hematoma that was seen on post-TAVR imaging. Given that the patient was asymptomatic, had no pericardial effusion, no involvement of the coronaries or sinus of Valsalva, and no evidence of aortic dissection, the authors in this case opted for conservative management with serial imaging. Follow-up imaging in this case demonstrated complete resolution of the intramural hematoma within 2 months of the TAVR procedure [12]. One case series described three patients who developed periaortic hematomas during a TAVR procedure. The authors identified several common features among these patients, which included female gender, advanced age, small body weight, mismatch of annulus and device diameter, and bulky calcification of the non-coronary cusp [13].

### 3.3. Aortic Fistula

Although rare, another possible iatrogenic aortic complication of TAVR is the formation of an aortic-cardiac fistula. Both aortic-ventricular and aortic-atrial fistulas have been described in the literature. One case described a small aorto-right ventricular fistula that was initially asymptomatic. This patient was followed with close monitoring, but symptoms of heart failure progressed. Repeat imaging demonstrated gradual enlargement of the fistula; however, given the patient’s frailty, no intervention was undertaken, and she eventually expired [14]. Another case of aorto-right ventricular fistula resulted in worsening heart failure, which prompted a discussion of possible treatment modalities. Ultimately, this patient underwent successful treatment with percutaneous closure using a septal occlusion device. This demonstrated that percutaneous management of this complication is a feasible option and potentially the preferred option for high-risk surgical patients [15]. Another case demonstrated a fistula between the aortic root and left atrium following TAVR. Because the patient developed symptoms, she was taken to the operating room for surgical repair with a Teflon strip reconstruction and a valve replacement [16]. A multicenter study examined all types of post-TAVR intra-cardiac shunts and found a rate of approximately 1.1% of this complication. Contributing factors may include porcelain aortas and treatment with pre- or post-dilation [17]. Other authors have similarly speculated that fistula formation is likely secondary to trauma from the displacement of heavily calcified aortic tissue [18].

### 3.4. Type A Aortic Dissection

Iatrogenic aortic dissections, as described in our case discussion above, are complications that can occur during open cardiac surgery, percutaneous coronary intervention, thoracic endovascular aortic aneurysm repair, and TAVR. One registry found that only 5% of all type A aortic dissections were iatrogenic in nature [19]. An analysis of the STS database revealed that the incidence of type A dissection during open cardiac surgery was 0.06% [20]. During cardiac catheterization procedures, the incidence is estimated to be 0.01–0.06% [21,22].

Although there have been several documented case reports of TAVR-associated ascending aortic dissection, it nonetheless remains a rare occurrence. Two single-center studies reported an incidence of aortic dissection of 0.1% [23] and 0.3% [24]. A meta-analysis of 16 studies demonstrated a reported rate of aortic dissection ranging from 0.9 to 1.7% with a pooled estimate rate of 1.1% [3]; and a study assessing nearly 16,000 TAVR procedures demonstrated an incidence of dissection to be 0.2% [25]. Based on this review of the literature, the incidence of dissection during TAVR procedures appears to be similar to that of both cardiac surgery and cardiac catheterization procedures.

Numerous mechanisms for the development of an aortic dissection during TAVR have been postulated. These include direct injury by stiff guidewire advancement, catheter injury to the aortic wall, and balloon valvuloplasty injury [24]. In a case series of TAVR-related dissections, the presumed causes were injury by delivery sheath in 39%, by delivery catheter in 23%, by valve implantation in 15%, by stent edge in 15%, and by pre-balloon dilation in 8% [26].

Aortic dissections have been demonstrated to occur more frequently with mechanically expandable valves when compared to self-expandable and balloon-expandable valves [11]. However, oversizing of a delivery balloon in a balloon-expandable valve may be an important risk factor for dissection, especially in patients with a narrow and calcified sinotubular junction [27]. For self-expanding valves, pre- and post-balloon dilation may also be a risk factor for dissection. One case report described the occurrence of an acute left main coronary artery dissection that occurred during post-dilation of a self-expandable valve. It was presumed in this case that calcifications cracked during dilation, leading to a dissection originating close to the ostium of the coronary artery [28]. Iatrogenic aortic-coronary dissections are known complications of interventional and diagnostic cardiac procedures; however, the overall risk of aortic-coronary dissection during TAVR is not well described in the literature [29].

Other researchers have sought to identify patient-related risk factors for dissection specifically associated with TAVR. Aortic wall weakening from steroids and immunologic medications, female sex, and atherosclerosis have all been cited as potential risk factors [30,31]. Kassis et al. also demonstrated a statistically significant increased risk of post-TAVR dissection in patients with a thoracic aortic aneurysm after controlling for bicuspid valve, gender, and comorbidities [31]. In a retrospective analysis of over one thousand TAVR patients, it was demonstrated that the chronic use of steroids pre-procedure conferred a higher risk of aortic complications (including aortic annular rupture, aortic dissection, and left ventricular perforation). In addition, this study demonstrated that steroid use was also associated with a higher rate of emergency conversion to open heart surgery. Interestingly, among patients with preoperative steroid use, there were no significant differences between balloon-expandable and self-expanding TAVR valves with respect to the primary outcome of aortic complications [32]. In our case described above, the patient had been on long-term steroids, which could contribute to aortic wall weakening and increase the susceptibility for aortic dissection. Additionally, the patient had previously been hospitalized with the COVID-19 infection, which has also been postulated to predispose patients to aortic wall weakening [33]. She did not, however, have any aneurysmal dilation or significant calcifications of her aorta on pre-TAVR imaging, which have been commonly cited as risk factors for many of the aortic complications discussed above.

The prevention strategies for TAVR-associated dissection and other aortic injuries can be broadly grouped into pre-procedural and procedural categories. In pre-procedure, the use of CT imaging is imperative to ascertain accurate measurements and to rule out specific anatomic risk factors such as tortuosity of the aorta, significant calcifications, and aneurysmal dilation that may confer a greater risk of dissection, as described above. During the procedure, it is important to consider valve size, sheath size, and proper catheter positioning during advancement and withdrawal. Post-procedural strategies should be aimed at prompt identification of the complication and the determination of appropriate management.

The management strategies of TAVR-associated iatrogenic aortic dissection are variable among cases reported in the literature. Although the procedure has been increasingly applied to low-risk surgical candidates, many of the patients who are selected for TAVR have comorbidities that place them at a prohibitive risk for surgical aortic valve replacement. As such, conversion to open surgery or “surgical bailout” after TAVR is often associated with an elevated risk of morbidity and mortality in these high-risk populations. In an analysis of the STS/ACC TVT registry, 1.17% of all TAVR procedures resulted in a complication requiring conversion to open surgery, and patients who underwent conversion had a significantly higher rate of all-cause hospital mortality compared to those who did not require conversion (49.64% vs. 3.52%). Aortic dissection represented 8.24% of the TAVR cases requiring surgical bailout in this study. Dissection was the fourth most common reason for conversion to open surgery, following ventricular rupture, prosthetic valve dislodgement, and annular rupture [10]. Similar results were described in a study that analyzed a European registry of TAVR procedures. This study reported that 11.8% of TAVR cases requiring emergent cardiac surgery were attributable to an aortic dissection, and the mortality rate among those patients was more than 50% [11]. In fact, von Aspern et al. demonstrated that in a single center, 50% of patients with TAVR-associated iatrogenic aortic dissection died within 30 days of the rescue surgery [23].

The in-hospital mortality rate among patients with a type A dissection who are managed non-operatively is estimated to be as high as 60% [34,35]. However, because of the high risk of mortality associated with conversion to open surgery, the decision to proceed to the operating room in such circumstances is based on patient and family preferences and a careful yet timely assessment of risks and benefits by the surgical team. After review of the current literature, many cases of TAVR-associated aortic dissection were ultimately treated non-operatively, either due to patient preference, severity of symptoms, or prohibitive surgical risk [24,36,37,38]. Some have demonstrated success with a conservative approach to type A dissection following TAVR followed by serial imaging [26,37,39,40]. There are also several documented cases of aortic dissection that have been amenable to thoracic endovascular aortic repair (TEVAR) [41,42,43,44]. Although endovascular management of type A dissections has demonstrated promising results, the data are limited to a small number of patients [45,46]. Furthermore, open surgical management remains the gold standard in the management of this devastating complication. This is especially true among patients in whom open surgical intervention is less likely to be futile and in those who can accept the high risks of surgery through an informed consent process.

The presence of cerebral malperfusion in the setting of a type A dissection is known to be a risk factor resulting in postoperative permanent neurologic deficits and even death. The management strategy for patients with dissection who present with evidence of cerebral malperfusion or coma has been controversial; however, several studies have supported surgical intervention as a lifesaving option in appropriate patients [47,48,49]. One study demonstrated that although patients with cerebral malperfusion demonstrated high postoperative hospital mortality, their long-term survival was similar to those without cerebral malperfusion (excluding preoperative coma patients) [50].

Imaging findings can also suggest the likelihood of poor neurologic recovery. One case series identified the presence of internal carotid artery (ICA) occlusion as a surrogate marker of devastating neurologic outcomes, including cerebral edema, herniation syndrome, and death [51]. In this study, 100% of patients with an ICA occlusion suffered neurologic death, regardless of management strategy. However, importantly, all patients in this series were transferred from outside hospitals, suggesting a prolonged time frame in this study from symptom onset to advanced imaging, diagnosis of aortic dissection, and management. Not unsurprisingly, time to surgery has been demonstrated consistently as a predictor of poor neurologic recovery following aortic repair for type A dissection [52]. One study demonstrated that the time from symptom onset to surgical repair was half as long in iatrogenic dissections compared to spontaneous dissections; however, iatrogenic dissections were associated with longer cardiopulmonary bypass and operative times. Interestingly, in this study, patients with post-interventional iatrogenic dissection (including TAVR, cardiac catheterization, and endovascular aortic repair) were more likely to undergo complex rescue surgery than patients with post-surgical iatrogenic type A dissection [19].

Despite the presence of neurologic symptoms suggesting cerebral malperfusion and imaging demonstrating ICA occlusion in our patient’s case, our use of conscious procedural sedation allowed for the prompt identification of severe chest pain and accurate assessment of neurologic status immediately following the TAVR procedure. This constellation of symptoms immediately raised concern for a possible acute aortic dissection. Urgent CT angiography demonstrated a dissection flap extending to the supra-aortic branches with no evidence of coronary obstruction or visceral involvement.

After proceeding to the operating room, the axillary arterial cannulation strategy was crucial for the success of this patient’s rescue surgery. A recent multicenter study examined surgical outcomes in patients with acute type A dissection and cerebral malperfusion and found that axillary arterial cannulation appeared to have a protective effect compared with femoral arterial cannulation. The authors presumed that antegrade systemic perfusion via the right axillary artery directly into the true lumen is effective at restoring normal flow to the brain. This study concluded that axillary cannulation may represent a preferred surgical strategy, especially among patients with a supra-aortic branch dissection [53]. This is further supported by a meta-analysis that demonstrated axillary arterial cannulation to be associated with fewer local and systemic complications, lower mortality, and fewer neurologic complications [54]. Fortunately, after initiating cardiopulmonary bypass in our patient, we elected to avoid direct antegrade cerebral perfusion through the right carotid artery given the immediate rise in cerebral oxygen saturation on the right side.

A postoperative diagnostic cerebral angiogram revealed no large vessel occlusion or non-flow-limiting dissection in the ICA. Fortunately, our patient did not require any further intervention based on these results, but we believe that timely evaluation by a stroke team and access to a neuro-interventional provider are important for the management of periprocedural stroke in TAVR patients. In fact, in an analysis of 11 cases of TAVR-associated stroke requiring mechanical thrombectomy, all patients demonstrated a significant reduction on the NIHSS scale. This led the authors of this study to propose that institutions implement dedicated pathways for TAVR-related stroke to promote improved outcomes [55].

The immediate diagnosis and decision to proceed to the operating room in our case of an aortic dissection were critical. Excellent communication and teamwork throughout this process contributed to a timely surgical bailout, leading to a positive outcome. The successful management of this potentially devastating TAVR complication further highlights the critical role that a multidisciplinary team at a specialized center plays in the era of transcatheter valve interventions. This case and our discussion of the literature highlight the importance of surgical backup for unexpected aortic complications of transcatheter procedures. Regardless of the type of injury, there are many common themes identified in the literature for the risk of aortic injury during TAVR. Common themes include heavy calcification seen on preoperative imaging, the use of pre- and post-dilation, valve oversizing, female sex, and pre-procedure steroid use. Despite these common themes, further research is needed to identify the patient-related risk factors and procedural mechanisms for TAVR-associated aortic injury and ways in which these risks can be mitigated moving forward.

## Data Availability

The data presented in this study are available on request from the corresponding author. The data are not publicly available due to patient privacy and confidentiality.
